# Efficacy of washed microbiota transplantation for therapeutic targets of refractory functional constipation and the influencing factors: a single-center, retrospective, 24-week follow-up study

**DOI:** 10.1186/s12876-023-02929-7

**Published:** 2023-08-28

**Authors:** Liquan Wu, Qingfen Yuan, Lihao Wu, Harry Hua-Xiang Xia, Muxiao Zhong, Tao Liu, Xiaoyan Ye, Danping Luo, Jiating Xu, Wenrui Xie, Xingxiang He, Jieyi Cai

**Affiliations:** 1 Department of Gastroenterology, Research Center for Engineering Techniques of Microbiota-Targeted Therapies of Guangdong Province, The First Affiliated Hospital of Guangdong Pharmaceutical University, No. 19 Nonglinxia Road, Yuexiu District, Guangzhou, 510080 China; 2 Department of Medical Record, The First Affiliated Hospital of Guangdong Pharmaceutical University, Guangzhou, 510080 China

**Keywords:** Fecal microbiota transplantation, Functional constipation, Therapeutic target, Clinical efficacy, Influencing factor

## Abstract

**Background:**

The efficacy of washed microbiota transplantation (WMT) in terms of refractory functional constipation (FC)-related therapeutic targets and influencing factors have not been elucidated. This study aimed to assess the efficacy and influencing factors of WMT in treating refractory FC-related therapeutic targets.

**Methods:**

The clinical data of patients diagnosed with refractory FC and received with WMT were retrospectively collected. The therapeutic targets included straining, hard stools, incomplete evacuation, a sense of anorectal obstruction, manual maneuvers, and decreased stool frequency. Each target was recorded as 1 (yes) or 0 (no). All patients were followed up for approximately 24 weeks from the end of the first course of WMT. The primary outcomes were the improvement rates for the individual therapeutic targets and the overall response in respect of the therapeutic targets decreased by 2 at weeks 4, 8, and 24. The secondary outcomes were the clinical remission rate (i.e., the proportion of patients with an average of 3 or more spontaneous complete bowel movements per week), clinical improvement rate (i.e., the proportion of patients with an average increase of 1 or more SCBMs/week or patients with remission), stool frequency, Wexner constipation score, Bristol Stool Form Scale (BSFS) score, and adverse events. The factors influencing the efficacy were also analyzed.

**Results:**

Overall, 63 patients with 112 WMT courses were enrolled. The improvement rates at weeks 8 and 24 were 45.6% and 35.0%, 42.9% and 38.6%, 45.0% and 35.7%, 55.6% and 44.4%, and 60.9% and 50.0%, respectively, for straining, hard stools, incomplete evacuation, a sense of anorectal obstruction, and decreased stool frequency. The overall response rates were 49.2%, 50.8%, and 42.9%, respectively, at weeks 4, 8, and 24. The rates of clinical remission and clinical improvement were 54.0% and 68.3%, respectively, at weeks 4. The stool frequency, BSFS score, and Wexner constipation score tended to improve post-WMT. Only 22 mild adverse events were observed during the 112 WMT courses and the follow-up. The number of WMT courses was identified to be the independent factor influencing the efficacy.

**Conclusions:**

WMT is efficacious in improving refractory FC-related therapeutic targets. The effectiveness of WMT in the management of FC is enhanced with the administration of multiple courses.

## Background

Functional gastrointestinal disorders, including functional constipation (FC), are highly prevalent worldwide, affecting more than 40% of the population, according to the first global epidemiological survey [1]. In adults, the estimated prevalence rate of FC is 11.7% worldwide and 10.6% in China [1], which has an adverse impact on the quality of life and brings about high healthcare costs [1, 2]. FC is symptom-based, non-organic in origin, and commonly diagnosed by Rome IV diagnostic [2, 3], and the symptoms of FC vary among different patients [4].

The pathophysiology of FC is considered to be multifactorial, and dysbiosis of gut microbiota is an important one of these factors. Although gut microbiota characteristics related to FC are inconsistent, emerging evidence [5–8] indicates that modulating the gut microbiota favors patients with FC. Fecal microbiota transplantation (FMT) is recognized as an emerging treatment through the reconstruction of gut microbiota, especially for refractory constipation [9]. Washed microbiota transplantation (WMT) is a microbiota transplantation method that is similar to traditional FMT but with a modification in that the washed microbiota prepared by an intelligent microorganism separation system, instead of the fecal microbiota, is used [10]. WMT has been proven to be superior to the fecal microbiota in the safety, quality control, and efficacy in the treatment of bacterial flora disorders [10]. Over the past few years, our team has observed that WMT is efficacious and safe for the treatment of several digestive and nondigestive diseases, including nonerosive reflux disease [11], *Helicobacter pylori* infection [12], chronic hemorrhagic radiation proctitis [13], gout [14], hypertension [15], dyslipidemia [16], and children with autism [17]. The therapeutic potential of FMT in refractory FC has recently been reported [18–21]. These studies demonstrated that FMT may relieve refractory FC by reconstructing gut microbiota and improving colonic transit time [6, 19–22]. However, it is difficult to quantitatively evaluate the overall efficacy of WMT for FC, and thus the primary efficacy outcomes in these studies mostly focused on decreased stool frequency but not on other equally important symptoms of FC, such as straining, hard stools, incomplete evacuation, a sense of anorectal obstruction and manual maneuvers [4]. Therefore, the efficacy of WMT in the treatment of these individual FC symptoms has not been explored. We postulate that WMT may be beneficial for most, if not all, refractory FC-related therapeutic targets, including straining, hard stools, incomplete evacuation, a sense of anorectal obstruction, manual maneuvers, and decreased stool frequency, in both efficacy and safety, but quantitative evaluation of the efficacy of WMT in terms of the individual therapeutic targets is required. In addition, there have been no studies exploring the potential factors influencing the efficacy of FMT or WMT for refractory FC. Therefore, the aim of this retrospective study was to assess the efficacy of and influencing factors for WMT in treating refractory FC-related therapeutic targets during a 24-week follow-up.

## Methods

### Participants and study design

This is a retrospective analysis of inpatients with refractory FC who received WMT. Refractory FC was defined as the situation where the patients failed to respond to the conventional or standard treatment [23]. The failure to respond to conventional or standard treatment was defined as follows: After strict lifestyle modifications and application of strong laxatives (e.g., prucalopride succinate) for 3 months, the patients still met the Rome IV chronic constipation diagnostic criteria [23]. Adult (> 18 years old) patients who presented with symptoms of chronic constipation for the last 3 months (with onset in the preceding 6 months) but without any organic gastrointestinal pathology, underwent WMT in our department from January 2017 to December 2019, and were regularly followed up for 24 weeks were eligible for inclusion. The symptoms of chronic constipation include two or more of the following based on the Rome IV criteria [23]: (i) straining: straining more than 25% of defecations, (ii) hard stool: lumpy or hard stools more than 25% of defecations, (iii) incomplete evacuation: a sense of sensation of incomplete evacuation more than 25% of defecations, (iv) a sense of anorectal obstruction: sensation of anorectal obstruction/blockage more than 25% of defecations, (v) manual maneuvers: manual maneuvers to facilitate more than 25% of defecations, and (vi) decreased stool frequency: fewer than three spontaneous complete bowel movements (SCBM) per week (Table 1). The exclusion criteria were as follows: patients with confirmed irritable bowel syndrome, pregnant patients, patients with incomplete clinical records and constipation secondary to drugs, patients with abuse history, or patients with endocrine or autoimmune diseases.

**Table 1:** Baseline definition of each therapeutic target and scoring method

	Scoring method	
Target	1	0
Straining^#^	Baseline: Straining more than 25% of defecations. Post-WMT: Frequency of straining unchanged or increased compared to baseline	(i) No straining (ii) Straining less than 25% of defecations.
Hard stools^#^	Baseline: Lumpy or hard stools more than 25% of defecations. Post-WMT: Frequency of hard stools unchanged or increased compared to baseline	(i) No hard stools (ii) Lumpy or hard stools less than 25% of defecations.
Incomplete evacuation^#^	Baseline: Sensation of incomplete evacuation more than 25% of defecations. Post-WMT: Frequency of incomplete evacuation unchanged or increased compared to baseline	(i) No straining (ii) Sensation of incomplete evacuation less than 25% of defecations.
Anorectal obstruction^#^	Baseline: Sensation of anorectal obstruction/blockage more than 25% of defecations. Post-WMT: Frequency of anorectal obstruction unchanged or increased compared to baseline	(i) No anorectal obstruction (ii) Sensation of anorectal obstruction/blockage less than 25% of defecations.
Decreased stool frequency^*^	Baseline: fewer than three spontaneous complete bowel movements per week. Post-WMT: Frequency of SCBMs/week unchanged or decreased compared to baseline	Three or more spontaneous complete bowel movements per week.

Definition: 1 = With any target at baseline; or frequency unchanged or increased^#^/ decreased^*^ post-WMT compared to baseline. 0 = Without target at baseline; or not matching any criteria compared to baseline

Decreased stool frequency, fewer than three spontaneous complete bowel movements per week; WMT, washed microbiota transplantation

Demographic and clinical data collected during hospitalization and follow-up visits were retrospectively retrieved from a computerized case record system established in our department. These data included age, sex, disease course of constipation, symptoms of constipation, Bristol Stool Form Scale (BSFS) score for stool consistency [24], Wexner score for the severity of constipation [25], the history of laxative use, delivery routes for WMT in the gut, number of courses of WMT treatment, and adverse events during WMT procedures. BSFS is a 7-point scale used extensively in clinical practice and research for stool form measurement [24]. Stools rated as scores 1 and 2 indicate constipation, and those rated as scores 6 and 7 indicate diarrhea. The Wexner constipation scale, consisting of questions on various clinical aspects of constipation, with scores ranging from 0 (best) to 30 (worst), is a validated and internationally adopted questionnaire used to quantify the severity of constipation [25]. A history of laxative use was defined as the situation when patients continuously used laxative drugs within 3 months before WMT. The delivery routes for WMT were transendoscopic enteral tubing (TET) or nasojejunal tube through mid-gut and colonic TET [26] through lower-gut, as previously described [26, 27], depending on the patient’s willingness and tolerance.

The study protocol was approved by the Ethics Committee of the First Affiliated Hospital of Guangdong Pharmaceutical University (No. 2020-11). All methods were carried out in accordance with relevant guidelines and regulations. All enrolled patients signed the informed consent for the WMT procedures.

### Donor screening and WMT procedures

All the patients signed an informed consent form prior to the WMT procedures, which were routinely carried out in hospitalized patients by using fresh microbiota at our hospital as previously described [15]. Briefly, healthy donor screening was performed with a questionnaire, and their stool and blood samples were tested to rule out potential infectious diseases or communicable diseases. A homogeneous fecal suspension of washed microbiota was prepared in a ratio of 100 g feces to 500 mL saline and then microfiltered (to remove fecal particles, parasite eggs, and fungi) by using an automatic machine (GenFMTer; FMT Medical, Nanjing, China). After microfiltration, the suspension was centrifuged at 1100 g for 3 min at room temperature. Then, the microbiota pellet was resuspended in saline. The centrifugation and resuspension of the microbiota pellet was repeated three more times. In the final resuspension, 100 mL saline was added to the microbiota pellet. Fecal suspensions from the various donors were randomly allocated to the patients. For the WMT procedure, the patient received the fecal suspension *via* mid-gut or through the lower digestive tract (120 mL per day for 3 consecutive days of one WMT course). All the doctors involved in this study had the same principle, that is, after a WMT course, if the patients still met the Rome IV criteria and the subjective feeling reported by the patient was not improved, then “no improvement” was defined. If the symptoms were improved or worsened, then “better’ or “worsen” was defined, respectively. The WMT procedure was repeated up to three times in patients with “no improvement” based on the doctor’s advice with the patient’s consent, with a 4-week interval between the first and second courses and between the second and third courses, and a 12-week interval between the third and fourth courses.

### Follow-up of patients after the first WMT procedure and evaluation of adverse events

Patients were followed up for approximately 24 weeks after the first course of the WMT procedure by regular hospital visits and telephone interviews. Changes in constipation symptoms, BSFS score, Wexner score, and the incidence of adverse events were monitored at weeks 4, 8, and 24.

Adverse events reported during the WMT treatment and the follow-up period were classified according to the Common Terminology Criteria for Adverse Events (version 5.0) [28] and graded as mild, moderate, or severe. Treatment-related adverse events were judged by the investigators. The relationship of the adverse events to WMT was evaluated.

### Assessment of efficacy and safety

The primary outcomes were the improvement for each of the therapeutic targets and the overall response with respect to the therapeutic targets at weeks 4, 8, and 24. The improvement of the individual therapeutic targets, including straining, hard stools, incomplete evacuation, a sense of anorectal obstruction, manual maneuvers, and decreased stool frequency, was assessed at each hospital visit or telephone call during the follow-up. The presence of each target was recorded as 1 (yes) or 0 (no) during the retrospective follow-up for each patient. The detailed definitions are listed in Table 1. The total score of the therapeutic targets was calculated by combining the score of each of the targets. Improvement of each of the therapeutic targets at weeks 4, 8, or 24 was defined if the target score changed from 1 at the baseline to 0 at the corresponding time point. Overall response at weeks 4, 8, or 24 was defined when the total scores of the therapeutic targets decreased by 2 at the corresponding time points, compared with the score at the baseline.

The secondary outcomes were (i) the clinical remission rate (i.e., the proportion of patients with an average of 3 or more SCBMs per week at weeks 4, 8, and 24); (ii) the clinical improvement rate (i.e., the proportion of patients with an average increase of 1 or more SCBMs/week or patients with remission at weeks 4, 8, and 24); (iii) the changes in the SCBMs per week, BSFS score, and Wexner score at weeks 4, 8, and 24; (iv) the changes in the proportions of patients with the therapeutic targets and those with BSFS score 1 and 2 at weeks 4, 8, and 24; and (v) the incidence of adverse events during the 24 weeks. We defined the clinical remission rate and the clinical improvement rate by SCBMs because this parameter has been used in many previous studies to evaluate the decreased stool frequency [6, 19–22].

### Identification of the potential factors influencing the efficacy of WMT in treating FC

Potential factors influencing the primary efficacy outcomes (i.e., the improvement of each of the therapeutic targets and the overall response) and secondary outcomes, including clinical remission and clinical improvement were performed according to the efficacy evaluated at weeks 4, 8, and 24. As delivery routes for WMT in patients receiving multiple courses of WMT were different, only data on the delivery route at week 4 were included in the analysis. In addition, data on the number of WMT courses were included in the analysis for the efficacy at weeks 8 and 24, but not at week 4, as all patients only completed the first course of WMT treatment at week 4.

### Statistical analysis

The continuous variables were expressed as a mean with standard deviation or a median with interquartile ranges (IQR), where appropriate. Qualitative variables were described as a percentage. To compare differences in continuous variables between groups, a *t*-test or rank-sum test was performed according to the data distribution. Chi-square test was used to analyze qualitative variables. The changes in the total score of targets between pre-WMT and post-WMT were analyzed using the Friedman test.

To identify the potential factors influencing the efficacy of WMT in treating FC, the associations of the primary and secondary outcomes with demographic and clinical parameters, including sex; age; number of SCBMs/week; BSFS score; Wexner constipation score; disease course of constipation; history of laxative use; delivery routes for WMT, including mid-gut (TET or nasojejunal tube through mid-gut [27]) and lower-gut (colonic TET [26]); and number of WMT courses were determined by univariate analyses, as described above. Then, parameters with a *P* < 0.1 were included in multivariate logistic regression analysis using a logistic regression method to identify independent factors influencing WMT efficacy. The odds ratio (OR) and 95% confidence interval (CI) were calculated. All statistical analyses were performed using SPSS 22.0 (IBM Corporation, Somers, NY, USA) and Prism 8 (GraphPad, San Diego, CA, USA). A *P*-value < 0.05 (two-tailed) was considered statistically significant.

## Results

### Patients’ characteristics

A total of 73 patients with refractory FC underwent WMT between January 2017 and December 2019; 65 patients were regularly followed up for 24 weeks and thus eligible for inclusion in the present study. Two patients were excluded due to incomplete clinical records (n = 2). Finally, 63 patients were included in the analyses. The baseline demographics and clinical characteristics of these patients are presented in Table 2.

**Table 2A:** Baseline characteristics and their associations with the improvement of therapeutic targets and the overall responses at week 4 in 63 refractory functional constipation patients treated with washed microbiota transplantation, as determined by univariate analyses

Baseline characteristics		Improvement of therapeutic targets and overall response^&^							
		Straining (26 vs. 34)	Hard stools (28 vs. 29)		Incomplete evacuation (6 vs. 8)	Anorectal obstruction (5 vs. 4)	Decreased stool frequency (26 vs. 20)	Overall response (31 vs. 32)	
Sex									
Male (n = 29)	46.0%	61.5% vs. 52.9%	53.6% vs. 55.2%		66.7% vs. 50.0%	80.0% vs. 75.0%	53.8% vs. 40.0%	61.3% vs. 46.9%	
Female (n = 44)	54.0%	38.5% vs. 47.1%	46.4% vs. 44.8%		33.3% vs. 50.0%	20.0% vs. 25.0%	46.2% vs. 60.0%	38.7% vs. 53.1%	
Age, year	60.8 ± 15.0	61.7 ± 12.1 vs. 59.5 ± 17.2	62.3 ± 11.7 vs. 58.9 ± 17.6		58.0 ± 15.9 vs. 67.5 ± 15.8	62.2 ± 7.7 vs. 71.5 ± 3.3	62.2 ± 12.2 vs. 59.0 ± 18.0	62.7 ± 12.5 vs. 60.0 ± 17.1	
Duration of disease, year	6 (2–12)	6.5 (2.0–20.0) vs. 6.5 (2.75–10.5)	7 (3-17.5) vs.6 (1–11)		2 (0.5–10) vs.7.5 (1.75–17.5)	3 (1.5–25) vs.6 (1.25–13.75)	8.5 (3-22.5) vs.6 (1–10)	7 (3–20) vs.6 (1–10)	
Number of SCBMs/week	2 (1–3)	5.0 (3.75–6.25) vs.2.5 (2.0–3.0)*	4 (3–6) vs.2 (2–3)*		6 (2.75-7) vs.3.5 (2.25–6.75)	5 (3-6.5) vs.4.5 (2.25–6.75)	3 (3–6) vs.2 (2–2)*	5 (3–6) vs.2 (2–3)*	
BSFS score	2 (1–2)	3.0 (3.0–4.0) vs.2.0 (2.0–3.0)*	3 (3-3.75) vs.2 (2–2)*		3 (3-4.25) vs.2 (2-3.75)	4 (2.5–4.5) vs.2.5 (1.25–3.75)	3 (2.75-4) vs.2 (2–2)*	3 (3–4) vs.2 (2–2)*	
Wexner constipation score	9 (8–11)	5 (3-7.25) vs.9 (8–10)*	6 (4–7) vs.10 (8-10.5)*		4 (2.75-6) vs.9 (7.25-10)*	7 (1.5-8) vs.8 (3.25–9.75)	7 (4.75-9) vs.9.5 (8–10)*	6 (4–8) vs.9 (8–10)*	
WMT delivery routes									
Mid-gut (n = 25) Lower-gut (n = 38)	39.7% 60.3%	34.6% vs. 41.2% 65.4% vs. 58.8%	32.1% vs. 48.3% 67.9% vs. 51.7%		50.0% vs.12.5% 50.0% vs.87.5%	40.0% vs. 25.0% 60.0% vs.75.0%	34.6% vs. 40.0% 65.4% vs.60.0%	32.3% vs. 46.9% 67.7% vs.53.1%	
History of laxative use	76.2%	61.5% vs. 85.3%*	71.4% vs.82.8%	66.7% vs. 87.5%		80.0% vs. 50.0%	76.9% vs. 80.0%	67.7% vs. 84.4%	

SCBMs/week, spontaneous complete bowel movements per week; WMT, washed microbiota transplantation; BSFS, Bristol Stool Form Scale;

&, responders vs. non-responders, responders were defined as those patients whose total scores decreased by 2 compared to the baseline;

#, one course of WMT was used as the reference, with which other courses were compared respectively,

Continuous data are presented as mean ± standard deviation or median with interquartile range, where appropriate, while categorical data are presented as number (percentage)

*, *P* < 0.05; **, *P* < 0.1

**Table 2B:** Baseline characteristics and their associations with the improvement of therapeutic targets and the overall responses at week 8 in 63 refractory functional constipation patients treated with washed microbiota transplantation, as determined by univariate analyses

Baseline characteristics		Improvement of therapeutic targets and overall response^&^							
		Straining (27 vs. 33)	Hard stools (26 vs. 31)		Incomplete evacuation (6 vs. 8)	Anorectal obstruction (5 vs. 4)	Decreased stool frequency (28 vs. 18)	Overall response (32 vs. 31)	
Sex									
Male (n = 29)	46.0%	55.6% vs. 57.6%	53.8% vs. 54.8%		66.7% vs. 50.0%	80.0% vs. 75.0%	50.0% vs. 44.4%	56.3% vs. 51.6%	
Female (n = 44)	54.0%	44.4% vs. 42.4%	46.2% vs. 45.2%		33.3% vs. 50.0%	20.0% vs. 25.0%	50.0% vs. 55.6%	43.8% vs. 48.4%	
Age, year	60.8 ± 15.0	61.3 ± 12.0 vs. 59.7 ± 17.4	61.5 ± 11.7 vs. 59.8 ± 17.4		58.0 ± 15.9 vs. 67.5 ± 15.8	62.2 ± 7.7 vs. 71.5 ± 3.3	61.7 ± 12.2vs. 59.5 ± 18.7	60.3 ± 12.1 vs. 61.1 ± 17.6	
Duration of disease, year	6 (2–12)	7 (2–15) vs. 6 (2.5–13.5)	10 (3–20) vs. 5 (1–10)		2 (0.5–10) vs. 7.5 (1.75–17.5)	3 (1.5–25) vs.6 (1.25–13.75)	7 (3-18.75) vs.6 (1–10)	7 (3-13.75) vs.6 (1–12)	
Number of SCBMs/week	2 (1–3)	5 (4–6) vs.3 (2–3)*	5 (3.75-6) vs.2 (2–3)*		5.5 (4–7) vs.3.5 (2.25–6.75)	4 (3–6) vs.4.5 (2.25–6.75)	4 (3-5.75) vs.2 (1–2)*	5 (3.25-6) vs.2 (2–3)*	
BSFS score	2 (1–2)	4 (3–4) vs.2 (2–3)*	3 (3–4) vs.2 (2–2)*		3.5 (3-4.25) vs.2 (2-3.75)**	3 (2.5–4.5) vs.2.5 (1.25–3.75)	3 (3–4) vs.2 (1.75-2)*	3.5 (3–4) vs.2 (2–2)*	
Wexner constipation score	9 (8–11)	6 (4–7) vs.10 (8–11)*	6 (4–7) vs.10 (8–11)*		5.5 (3–8) vs.9 (6.5–10)**	7 (3.5–12.5) vs.7.5 (3.25–9.5)	6 (5.25–9.5) vs.10 (8-10.25)*	6 (4–7) vs.10 (8–11)*	
Course of WMT	1 (1–3)	2 (1–3) vs.1 (1–2)*	2 (1–3) vs.1 (1–2)*		3 (1–3) vs.1 (1–2)	1 (1–3) vs.1 (1-1.75)	2 (1–3) vs.1 (1–1)*	2 (1–3) vs.1 (1–1)*	
1 (n = 37)^#^	58.7%	40.7% vs. 72.7%	46.2% vs. 74.2%		33.3% vs. 62.5%	60.0% vs. 75.0%	32.1% vs. 94.4%	37.5% vs. 80.6%	
2 (n = 10)	15.9%	22.2% vs. 9.1%**	19.2% vs. 12.9%		0.0% vs. 25.0%	0.0% vs. 25.0%	21.4% vs. 5.6%*	25.0% vs. 6.5%*	
3 (n = 16)	25.4%	37.0% vs. 18.2%*	34.6% vs. 12.9%*		66.75 vs. 12.5%	40.0% vs. 0.0%	46.4% vs. 0.0%	37.5% vs. 12.9%*	
Course of WMT ≥ 2 (n = 26)	41.3%	59.3% vs. 27.3%*	53.8% vs. 25.8%*	66.7% vs. 37.5%		40.0% vs. 25.0%	67.9% vs. 5.6%*	62.5% vs. 19.4%*	
History of laxative use	76.2%	63.0% vs. 84.8%**	73.1% vs. 80.6%	66.7% vs. 87.5%		80.0% vs. 50.0%	75.0% vs. 83.3%	68.8% vs. 83.9%	

SCBMs/week, spontaneous complete bowel movements per week; WMT, washed microbiota transplantation; BSFS, Bristol Stool Form Scale;

&, responders vs. non-responders, responders were defined as those patients whose total scores decreased by 2 compared to the baseline;

#, one course of WMT was used as the reference, with which other (i.e., two, three, and two plus three) courses were compared, respectively;

Continuous data are presented as mean ± standard deviation or median with interquartile range, where appropriate, while categorical data are presented as number (percentage)

*, *P* < 0.05; **, *P* < 0.1

**Table 2C:** Baseline characteristics and their associations with the improvement of therapeutic targets and the overall responses at week 24 in 63 refractory functional constipation patients treated with washed microbiota transplantation, as determined by univariate analyses

**Baseline characteristics**		Improvement of therapeutic targets and overall response^&^					
		Straining (21 *vs*. 39)	Hard stools (22 *vs*. 35)	Incomplete evacuation (5 *vs*. 9)	Anorectal obstruction (4 *vs*. 5)	Decreased stool frequency (23 *vs*. 23)	Overall response (27 *vs*. 36)
Sex							
Male (n = 29)	46.0%	57.1% *vs.* 56.4%	50.0% *vs.* 57.1%	60.0% *vs.* 55.6%	75.0% *vs.* 80.0%	52.2% *vs.* 43.5%	55.6% *vs.* 52.8%
Female (n = 44)	54.0%	42.9% *vs.* 43.6%	50.0% *vs.* 42.9%	40.0%*vs.* 44.4%	25.0% *vs.* 20.0%	47..8% *vs.* 56.5%	44.4% *vs.* 47.2%
Age, year	60.8 ± 15.0	62.0 ± 10.7 *vs.* 60.0 ± 17.1	61.0 ± 11.6 *vs.* 60.3 ± 17.0	62.8 ± 12.0 *vs.* 63.8 ± 6.2	59.3 ± 4.6 *vs.* 72.0 ± 3.1	60.7 ± 11.0 *vs.*61.0 ± 18.3	61.4 ± 10.9 *vs.* 60.4 ± 17.5
Duration of disease, year	6 (2–12)	10 (4-12.5) *vs.* 6 (1–15)	10 (4.5–20) *vs.* 5 (1–10)	3 (0.5–10) *vs.* 5 (1–15)	11.5 (2.25–27.5) *vs.* 2 (1-12.5)	10 (5–20) *vs.* 5 (1–10)**	10 (3–15) *vs.* 5 (1-11.5)
Number of SCBMs/week	2 (1–3)	5 (4–6) *vs.* 2 (2–3)*	5 (3-5.25) *vs.* 2 (2–3)*	5 (3-6.5) *vs.* 4 (2–7)	4.5 (2.5–6.5) *vs.*3 (2-6.5)	4 (3–5) *vs.* 2 (2–2)*	5 (3–6) *vs.* 2 (2–3)*
BSFS score	2 (1–2)	3 (3–4) *vs.* 2 (2–3)*	3 (3–4) *vs.* 2 (2–2)*	3 (2.5-4) *vs.* 2 (1.5–4.5)	3.5 (2.25–4.75) *vs.* 2 (1.5–3.5)	3 (3–4) *vs.* 2 (2–2)*	3 (3–4) *vs.* 2 (2–2)*
Wexner constipation score	9 (8–11)	6 (3–7) *vs.* 9 (8–10)*	6 (3-7.25) *vs.* 9 (8–10)*	6 (3-9.5) *vs.* 9 (6–10)	9 (2.5-11.75) *vs.* 8 (4.5–9.5)	7 (5–9) *vs.* 9 (8–10)*	6 (3–8) *vs.* 9 (8–10)*
Course of WMT	1 (1–3)	2 (1-2.5) *vs.* 1 (1–3)	1.5 (1–3) *vs.* 1 (1–2)	3 (1-3.5) *vs.* 1 (1-2.5)	1 (1-2.5) *vs.* 1 (1-2.5)	2 (1–3) *vs.* 1 (1–2)*	2 (1–3) *vs.* 1 (1–2)*
1 (n = 37)^#^	58.7%	47.6% *vs.* 64.1%	50.0% *vs.* 68.6%	40.0% *vs.* 55.6%	75.0% *vs.* 60.0%	39.1% *vs.* 73.9%	40.7% *vs.* 72.2%
2 (n = 10)	15.9%	28.6% *vs.* 7.7%*	22.7% *vs.* 11.4%	0.0% *vs.* 22.2%	0.0% *vs.* 20.0%	17.4% *vs.* 13.0%	25.9% *vs.* 8.3%*
3 (n = 9)	14.3%	4.8% *vs.* 20.5%	9.1% *vs.* 14.3%	40.0% *vs.* 11.1%	25.0% *vs.* 20.0%	26.1% *vs.* 13.0%	14.8% *vs.* 13.9%
4 (n = 7)	11.1%	19.0% *vs.* 7.7%	18.2% *vs.* 5.7%	20.0% *vs.* 11.1%	0.0% *vs.* 0.0%	17.4% *vs.* 0.0%	18.5% *vs.* 5.6%**
Course of WMT ≥2 (n = 26)	41.3%	52.4% *vs.* 35.9%	50.0% *vs.* 31.4%	60.0% *vs.* 44.4%	25.0% *vs.* 40.0%	60.9% *vs.* 26.1%*	59.3% *vs.* 27.8%*
History of laxative use	76.2%	57.1% *vs.* 84.6%*	72.7% *vs.* 80.0%	60.0% *vs.* 88.9%	75.0% *vs.* 60.0%	78.3% *vs.* 78.3%	70.4% *vs.* 80.6%

SCBMs/week, spontaneous complete bowel movements per week; WMT, washed microbiota transplantation; BSFS, Bristol Stool Form Scale;

&, responders vs. non-responders, responders were defined as those patients whose total scores decreased by 2 compared to the baseline;

#, one course of WMT was used as the reference, with which other (*i.e.*, two, three, four and two plus three plus four) courses were compared, respectively;

Continuous data are presented as mean ± standard deviation or median with interquartile range, where appropriate, while categorical data are presented as number (percentage)

*, *P* < 0.05; **, *P* < 0.1

Of these patients, a total of 112 courses of WMT were performed; 37, 10, 9, and 7 patients underwent 1, 2, 3, and 4 courses, respectively. The lower-gut delivery route was preferred by 60.3% (38/63), 34.6% (9/26), 43.8% (7/16), and 42.9% (3/7) of patients in the first, second, third, and fourth courses, respectively.

### Primary efficacy outcomes of WMT for individual therapeutic targets of FC

**Fig. 1:**
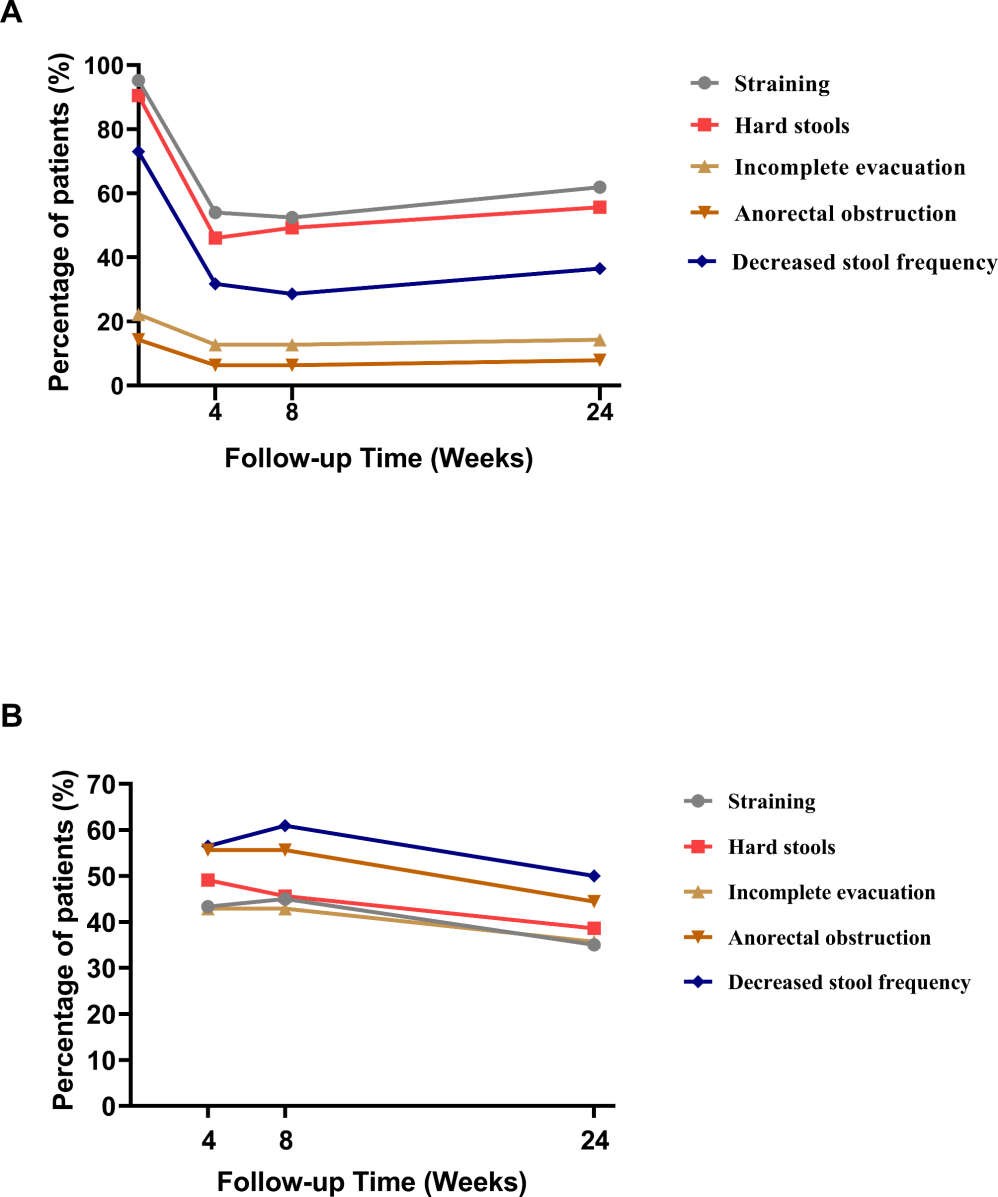
Changes in the proportions of patients with the five individual therapeutic targets **(A)** and the proportions of patients with improvement of the five individual therapeutic targets **(B)** during the 24-week follow-up after washed microbiota transplantation

Of the 63 patients with FC, 95.2% (n = 60), 90.5% (n = 57), 22.2% (n = 14), 14.3% (n = 9), and 73.0% (n = 46) complained of straining, hard stools, incomplete evacuation, a sense of anorectal obstruction, and decreased stool frequency, respectively; none complained of manual maneuvers at the baseline (Fig. 1A). The proportions of patients with these five individual therapeutic targets were reduced to 54.0% (n = 34), 46.0% (n = 29), 12.7% (n = 8), 6.3%(n = 4), and 31.7% (n = 20) at week 4; 52.4% (n = 33), 49.2% (n = 31), 12.7% (n = 8), 6.3% (n = 4), and 28.6% (n = 16) at week 8; and 61.9% (n = 39), 55.6% (n = 35), 14.3% (n = 9), 7.9% (n = 5), and 36.5% (n = 23) at week 24, respectively. The proportions of patients with straining, hard stools, and decreased stool frequency were significantly decreased at weeks 4, 8, and 24 post-WMT (vs. Pre-WMT), respectively (all *P* < 0.001, r=-0.240, r=-0.232, and r=-0.263, respectively) (Fig. 1A). However, there was no significant change in the proportion of patients with incomplete evacuation (*P* = 0.391, r=-0.074) or sense of anorectal obstruction (*P* = 0.336, r=-0.075) at weeks 4, 8, and 24 post-WMT (vs. Pre-WMT). The median total score of the individual therapeutic targets before WMT was 3 (range, 2–5), which significantly decreased to 1 (range, 0–4), 1 (range, 0–4), and 1 (range, 0–3), respectively, at weeks 4, 8, and 24 (all *P* < 0.001).

At week 4, 43.3%, 49.1%, 42.9%, 55.6%, and 56.6% of patients achieved improvement in straining, hard stools, incomplete evacuation, a sense of anorectal obstruction, and decreased stool frequency, respectively, with the improvement rates for decreased stool frequency and a sense of anorectal obstruction being the highest amongst the five targets (Fig. 1B). The proportions of patients with improvement of the five therapeutic targets were 45.6%, 42.9%, 45.0%, 55.6%, and 60.9% at week 8 and 38.6%, 35.7%, 35.0%, 44.4%, and 50.0% at week 24, respectively (Fig. 1B).

In addition, the overall response rates were 49.2% (n = 31), 50.8% (n = 32), and 42.9% (n = 27), respectively, at weeks 4, 8, and 24.

### Secondary efficacy and safety outcomes of WMT for FC

Both clinical remission and improvement rates reached their highest level at week 4, with rates of 54.0% (n = 34) and 68.3% (n = 43), respectively, which were then slightly reduced to 54.0% (n = 34) and 63.5% (n = 40) at week 8 and 44.4% (n = 28) and 57.1% (n = 36) at week 24. There were no significant differences in the rates among different follow-up time points.

**Fig. 2:**
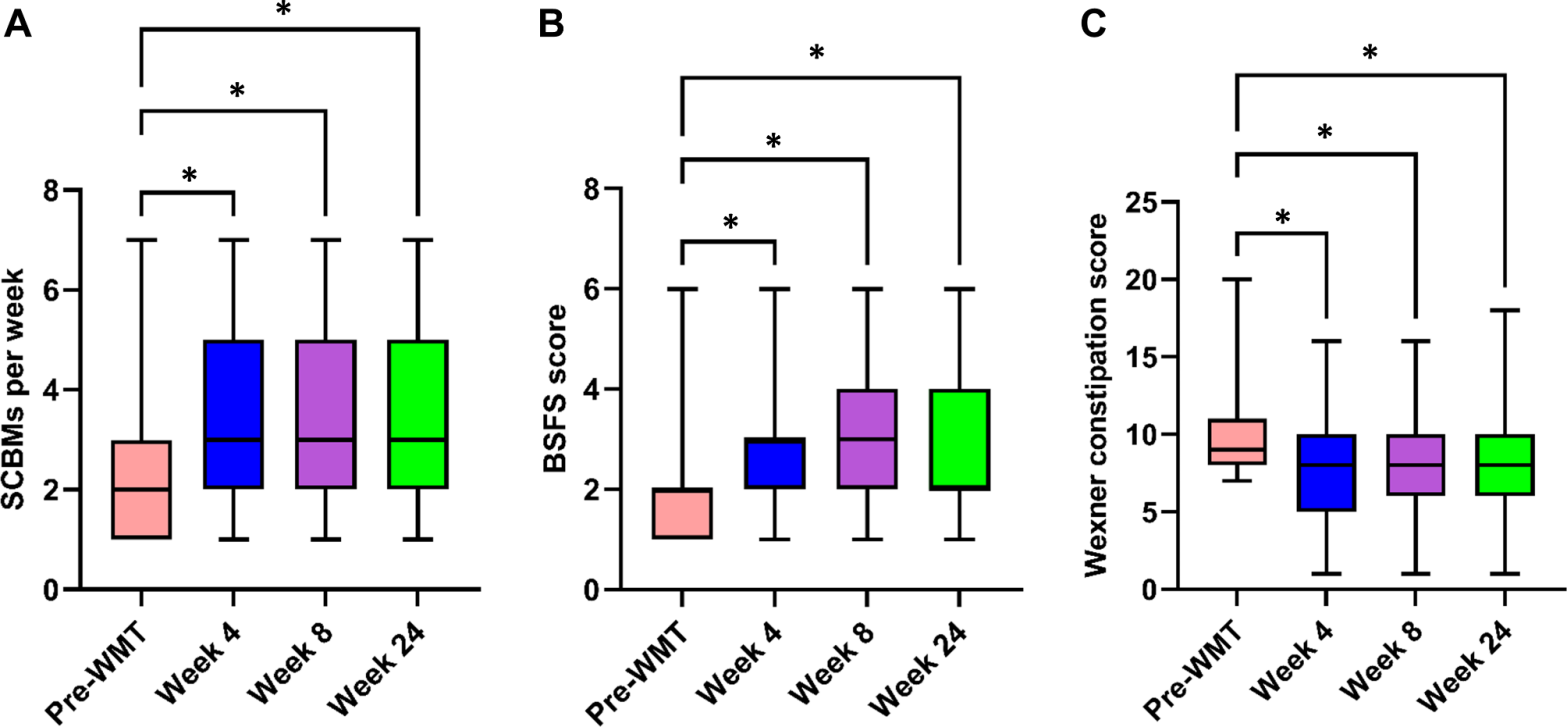
Therapeutic efficacy of washed microbiota transplantation (WMT) for functional constipation as determined by spontaneous complete bowel movements (SCBMs) per week **(A)**, Bristol Stool Form Scale (BSFS) score **(B)**, and Wexner constipation score (**C**) Data are expressed as median (interquartile range); * *P* < 0.001, compared with Pre-WMT.

WMT also exhibited a beneficial therapeutic efficacy in treating FC, as determined by the changes in SCBMs per week, BSFS score, and Wexner constipation scores, which demonstrated a significant time-by-treatment interaction in the constipation-related symptom scores (Fig. 2). Specifically, the SCBMs per week increased from 2 (range, 1 to 3) per week pre-WMT to 3 (range, 2 to 5), 3 (range, 2 to 5), and 3 (range, 2 to 5) per week, respectively, at weeks 4, 8, and 24 without laxative use (all *P* < 0.001, Fig. 2A). The BSFS score increased from 2 (range, 1 to 2) pre-WMT to 3 (range, 2 to 3), 3 (range, 2 to 4), and 2 (range, 2 to 4), respectively, at weeks 4, 8, and 24 (all *P* < 0.001, Fig. 2B). Furthermore, the Wexner score significantly reduced from 9 (range, 8 to 10) before WMT to 8 (range, 5 to 10), 8 (range, 6 to 10), and 8 (range, 6 to 10), respectively, at weeks 4, 8, and 24 (all *P* < 0.001, Fig. 2C).

**Fig. 3:**
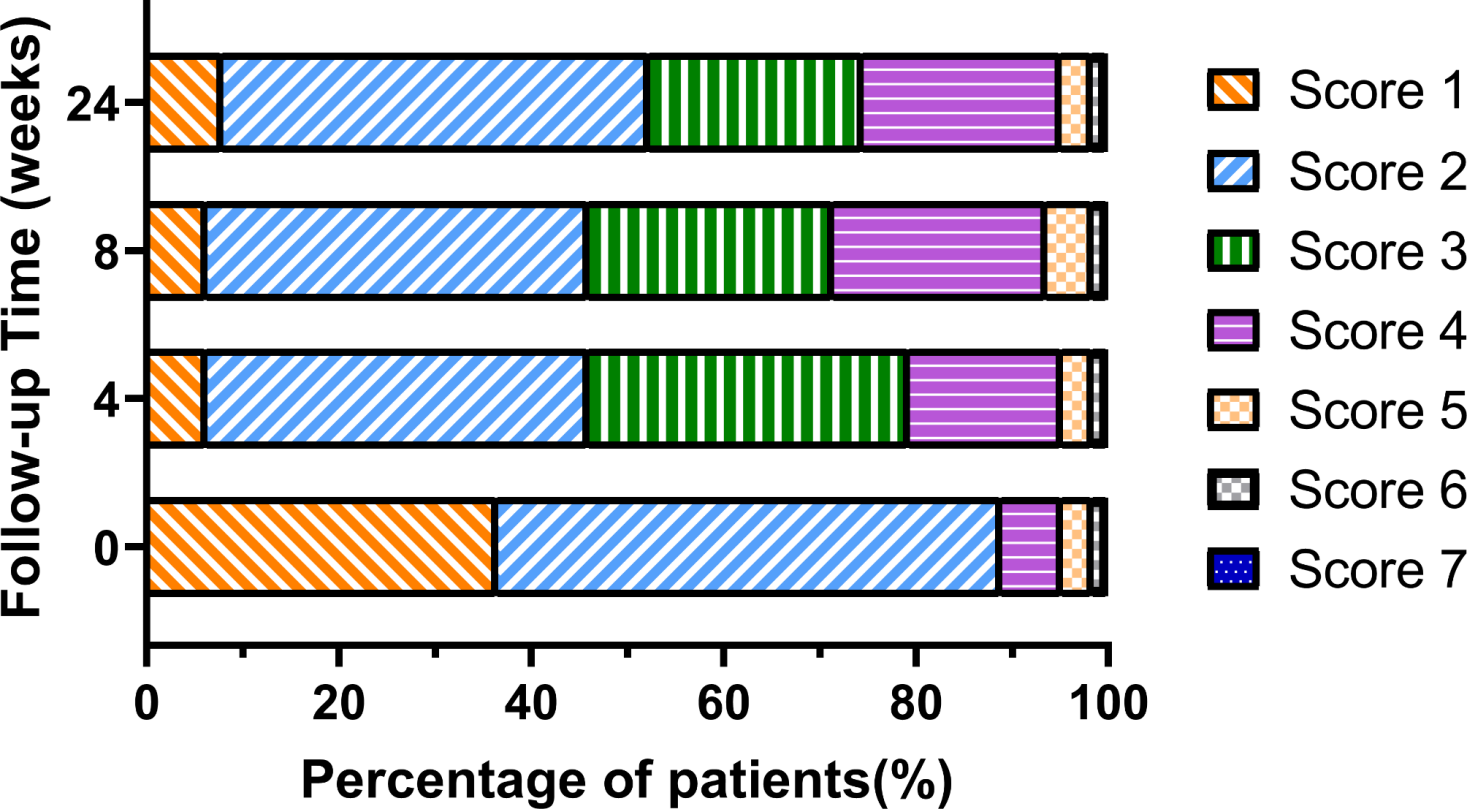
Changes in the proportions of Bristol Stool Form Scale score in patients with functional constipation at baseline and 4, 8, and 24 weeks after washed microbiota transplantation

The changes in the proportion of patients with various BSFS scores are depicted in (Fig. 3**)**. At week 4 post-WMT, the proportions of patients with BSFS score 1 decreased from 36.5 to 6.3% (*P* < 0.001). However, the proportions of patients with BSFS score 2 decreased from 52.4 to 39.7% (*P* = 0.153); the proportions appeared to be stable at weeks 8 and 24.

A total of 112 WMT procedures were performed in the 63 patients. Overall, eight (12.7%) patients experienced 22 adverse events: three patients experienced one, and five patients experienced more than one adverse event. The incidence was 19.6 per 100 WMT procedures. All these adverse events were judged by the investigators to be related to the WMT treatment procedures. Diarrhea, abdominal pain, and bloating were the most common treatment-related adverse events, with the rates being 7.0%, 3.6%, and 3.6%, respectively, during the days of WMT procedure, and 0.9%, 0.9%, and 1.8% during the follow-up (Table 3). All the adverse events were mild and resolved with self-limiting. No serious adverse events were observed.

**Table 3:** Treatment-related adverse events observed during the washed microbiota transplantation procedure and 24-week follow-up

Adverse event*	On days of infusion	During follow-up
Fever	0 (0.0)	0 (0.0)
Diarrhea	8 (7.0)	1 (0.9)
Abdominal pain	4 (3.6)	1 (0.9)
Nausea	1 (0.9)	0 (0.0)
Increased bloating	4 (3.6)	2 (1.8)
Vomiting	1 (0.9)	0 (0.0)
Overall	18	4

*, a total of 112 washed microbiota transplantation procedures were performed in the 63 patients with functional constipation. One patient may experience more than one adverse event

Categorical data are presented as number (percentage)

### Factors influencing the efficacy of WMT treatment

In the univariate analyses, a history of laxative use and the number of courses of WMT were significantly associated with the improvement of one of the therapeutic targets and the overall response at weeks 4 (Table 2 A), 8 (Table 2B), and 24 (Table 2 C). In the multivariate analysis in which all factors with a *P* < 0.1 in the univariate analyses were included, the course of WMT (≥ 2 courses vs. 1 course) was independently associated with the improvement of straining (OR = 4.458, 95% CI: 1.412–14.075, *P* = 0.011), hard stools (OR = 3.354, 95% CI: 1.101–10.222, *P* = 0.033), decreased stool frequency (OR = 35.889, 95% CI: 4.109-313.423, *P* = 0.001), and overall response (OR = 6.944, 95% CI: 2.215–21.776, *P*＜0.001) at week 8 and with the improvement of decreased stool frequency (OR = 4.897, 95% CI: 1.322–18.139, *P* = 0.017) and overall response (OR = 3.782, 95% CI: 1.312–10.903, *P* = 0.014) at week 24 (Table 4). Moreover, the straining improvement rates at weeks 4 and 24 were significantly higher in patients without a history of laxative use at the baseline than in those with the history (66.7% (10/15) vs. 35.6% (16/45), χ^2^ = 4.434, *P* = 0.035 and 60.0% (9/15) vs. 26.7% (12/45), χ^2^ = 8.595, *P* = 0.019, respectively). In the multivariate analysis, a history of laxative use was the independent factor associated with the straining improvement at week 4 (OR = 0.276, 95% CI: 0.080–0.948, *P* = 0.041), week 8 (OR = 0.251, 95% CI: 0.066–0.944, *P* = 0.041), and week 24 (OR = 0.227, 95% CI: 0.064–0.798, *P* = 0.021). We further conducted a factor analysis on the use of laxative drugs and found that patients with higher Wexner constipation scores were more inclined to use laxatives at the baseline (OR = 1.836, 95% CI: 1.101–3.063, *P* = 0.020). Moreover, the Wexner constipation scores were significantly associated with therapeutic targets such as straining, hard stools, and decreased stool frequency (Table 2 A-C).

**Table 4:** Influence of the courses of washed microbiota transplantation on major primary and secondary outcomes in functional constipation patients at weeks 8 and 24, as determined by multivariate logistic regression analysis

Variable	At week 8		At week 24	
	Odds ratio (95% CI)	*P*-value	Odds ratio (95% CI)	*P*-value
*Primary outcomes*				
Straining improvement rate	4.458 (1.412–14.075)	0.011	2.177 (0.693–6.836)	0.183
Hard stools improvement rate	3.354 (1.101–10.222)	0.033	2.182 (0.727–6.548)	0.164
Decreased stool frequency improvement rate Overall response	35.889 (4.109-313.423) 6.944 (2.215–21.776)	0.001 0.001	4.897 (1.322–18.139) 3.782 (1.312–10.903)	0.017 0.014
*Secondary outcomes*				
Clinical remission rate	4.571 (1.222–17.097)	0.024	2.109 (0.558–7.975)	0.272
Clinical improvement rate	10.214 (1.398–74.623)	0.022	5.668 (1.436–22.363)	0.013

WMT, washed microbiota transplantation; CI, confidence interval. Decreased stool frequency, fewer than three spontaneous complete bowel movements per week;

Comparison between two or more courses versus one course of WMT.

In addition, the clinical remission and improvement rates were significantly higher in patients receiving ≥ 2 courses of WMT treatment than in those receiving one course at week 8 (80.8% (21/26) vs. 35.1% (13/37), χ^2^ = 12.800, *P* < 0.001 and 92.3% (24/26) vs. 43.2% (16/37), χ^2^ = 15.858, P < 0.001, respectively) and week 24 (57.7% (15/26) vs. 35.1% (13/37), χ^2^ = 3.147, *P* = 0.076 and 80.8% (21/26) vs. 40.5% (15/37), χ^2^ = 10.091, *P* = 0.001 respectively). In the multivariate analysis, the course of WMT treatment (≥ 2 courses vs. 1 course) was the only independent factor associated with the clinical remission (OR = 4.571, 95% CI: 1.222–17.097, *P* = 0.024) and clinical improvement (OR = 10.214, 95% CI: 1.398–74.623, *P* = 0.022) at week 8 and with the clinical improvement (OR = 5.668, 95% CI: 1.436–22.363, *P* = 0.013 at week 24).

## Discussion

In the present study, WMT treatment significantly improved FC-related therapeutic targets, especially straining, hard stools, and decreased stool frequency at weeks 4, 8, and 24 after the treatment, with the improvement rates and the overall response rates being the highest at week 8. Moreover, patients with more than one course of WMT responded better to WMT with respect to the improvement of the therapeutic targets at weeks 8 and 24, and patients without a history of laxative use at the baseline responded better to WMT with respect to the improvement of straining at weeks 4 and 24. In addition, clinical remission and improvement rates were the highest at week 4. The stool frequency, BSFS score, and Wexner constipation score all tended to improve post-WMT. Only 22 mild adverse events were observed during the 112 WMT courses and the follow-up in the 63 patients.

Currently, FC is generally managed with various medicines and dietary supplements [4], which are either inefficacious or associated with unwanted adverse effects, including abdominal distension, abdominal pain, diarrhea, flatulence, nausea, vomiting, and headache [29]. Recently, FMT has been reported as a promising modality for the treatment of FC by restoring a healthy gut microbiota with fewer adverse events [20]. In the present study, we specifically evaluated the efficacy of WMT in treating refractory FC, with a focus on FC-related therapeutic targets, including straining, hard stools, incomplete evacuation, manual maneuvers, a sense of anorectal obstruction, and decreased stool frequency. As no patients complained of manual maneuvers in the present study, we evaluated the efficacy of WMT for treating the other five therapeutic targets. At present, only some studies explore the efficacy of WMT on decreased stool frequency [18–21], and the efficacy of WMT on other four therapeutic targets (straining, hard stools, incomplete evacuation, and sense of anorectal obstruction) remains largely unknown.

In the present study, the most frequent complaints were straining (95.2%) and hard stools (90.5%), which are consistent with the findings of the previous study (81.0% and 72.0%, respectively, for straining and hard stools) [30]. We observed that WMT treatment improved all five FC-related therapeutic targets, especially for straining, hard stools, and decreased stool frequency during the 24-week follow-up study; improvement in straining and hard stools was achieved in more than 40% (43.3% and 49.1%, respectively) of patients at week 4 after the first WMT. However, the rates tended to decline at week 24 of follow-up, suggesting that this therapeutic effect may not bring long-term benefits for these two therapeutic targets.

Our findings revealed that 56.6% of patients achieved improvement in decreased stool frequency at week 4 after the first WMT. The rate increased to 60.9% at week 8 and stabilized at 50.0% at week 24. In the present study, the rates of overall clinical improvement and clinical remission were 68.3% and 54.0%, 63.5% and 54.0%, and 57.1% and 44.4%, respectively, at weeks 4, 8, and 24. In the study by Zhang et al. [18], after the one year follow up, 48.3% (14/29) of patients continued to have at least three complete spontaneous bowel movements per week, and 58.6% (17/29) of patients showed clinical improvements. The high positive response in their study may be due to the fact that the patients received 6-day FMT procedures repeatedly for the first 3 months and soluble dietary fiber (pectin) daily during the follow-up. This treatment intensity was higher than what we did. In addition, we do not know whether the patients in our study can still achieve clinical improvements after one year. In the study by Ding et al. [19], after FMT, the percentage of patients achieving primary efficacy endpoint (at least three CSBMs per week) over the week intervals 3–4, 9–12, and 21–24 increased to 50.0%, 38.5%, and 32.7%, respectively. The results were lower than the results in our study. In the study by Tian et al. [20], the clinical improvement rate was 53.3%, which was similar to the results in our study. In another study by Tian et al. [21], the rates of clinical improvement and remission based on clinical activity at week 12 were 50% (12/24) and 37.5% (9/24), respectively, which were lower than the rates in our study. Xie et al. [22] concluded that clinical improvement and clinical remission reached 62.5% and 75%, respectively, after the third treatment. However, no follow up data is available in their study. The combination medication with FMT was not specified in their studies. It seems that only FMT was used as the treatment method in previous studies. In general, we believe the results in our study were comparable to the previous studies [19–22], which showed that FMT increased the stool frequency with high clinical remission rate and clinical improvement rates in patients with FC. It has been shown that the improvement of the therapeutic target of decreased stool frequency by FMT is correlated with the decreased of the colonic transit time [21, 31, 32]. Furthermore, we investigated the change in stool consistency and compared pre-WMT with post-WMT using the BSFS score, which may reflect fecal water content and activity and is considered a substitute index for intestinal colon transit time [33]. The increased BSFS score in our study demonstrated that stool consistency has a significant improvement post-WMT. It is known that gut microbiota disturbance exists in patients with constipation and plays a key role in disturbing colonic motility. Recently, several studies [6, 33–37] have demonstrated a correlation between stool consistency and several members of the fecal microbiota composition, such as the *Bacteroidetes* to *Firmicutes* ratio and the abundance of *Bifidobacterium*, *Fusicatenibacter*, *Paraprevotella*, and *Lachnoanaerobaculum*. These findings indicate that WMT relieves constipation symptoms by remodeling the gut microbiota composition. We postulate that the possible mechanism might involve alterations in the abundances of key bacteria, metabolism of products such as short-chain fatty acids [6, 38], and serum concentrations of cytokines, such as interleukin-8 [6], interleukin-6 [39, 40], and interleukin-12 [39], which result in motility changes and consequently, the improvement of the stool frequency and consistency [6].

At present, attempts to treat patients with anorectal obstruction feelings not caused by mechanical obstruction are biofeedback therapy and rectal administration of laxatives or transanal irrigation [41]. Yang et al. reported that WMT combined with biofeedback had sustained efficacy with similar adverse events compared with biofeedback therapy alone in patients with mixed constipation, suggesting that WMT may upregulate the beneficial bacteria and reshape the composition of intestinal microbiota in patients with mixed constipation and explaining the efficacy of WMT [42]. The present study demonstrated that 55.6% (5/9) of patients with a sense of anorectal obstruction achieved symptom improvement at week 4 after the first WMT course, supporting its therapeutic effect on the FC symptom of a sense of anorectal obstruction.

The current delivery routes for WMT in the gut include the upper gut (*via* oral capsule, or drink), mid gut (*via* gastroscopy in the duodenum, mid-gut tubing, or PEG-J tube), and lower gut (*via* colonoscopy, colonic transendoscopic enteral tubing, traditional enema, or stoma in the ilecolon) [43]. There have been some studies to evaluate the various routes of WMT administration in *Clostridioides difficile* infection [44, 45] However, there is no reported difference in the efficacy among the different delivery routes for the treatment of FC. In the present study, we found no significant difference in the improvement of therapeutic targets and overall response at week 4 between mid-gut and lower-gut routes. As reported, both mid-gut TET and colonic TET through lower-gut are novel, convenient, reliable, and safe procedures for microbiota transplantation with a high degree of patient satisfaction [26, 27, 43], and colonic TET is recommended for patients who need frequent WMTs or WMT combined with other medications^43^. For each delivery route, many factors should be considered, including aesthetics, psychology, and privacy [43].

Repeated FMT strategies have been employed with success to a certain extent in a number of different clinical entities, such as refractory constipation [46], irritable bowel syndrome [47], refractory *Clostridioides difficile* infection [48, 49], and inflammatory bowel disease [50]. In the present study, repeated WMT courses increased the overall response rate, the improvement rates for most FC-related therapeutic targets, and the clinical remission and improvement rates, indicating that multiple courses of WMT are required to consolidate the efficacy of WMT. At present, there are few studies on the mechanism of repeated WMT. Mocanu et al. [50] demonstrated that repeated FMT courses provide a promising approach to improve inflammatory bowel disease outcomes by increasing fecal microbiota richness, α-diversity, and several SCFA-producing anaerobic taxa. However, further standardization of WMT therapies is required to bring microbial-targeted therapies based on WMT from basic research to clinical practice [50].

We found that, interestingly, patients with higher Wexner constipation scores at the baseline were more likely to use laxative drugs, and patients with a history of laxative use responded worse to WMT on the improvement of straining. The Wexner constipation scale is used to quantify the severity of constipation [25]. It is conceivable that patients with more severe constipation symptoms would have a worse response to WMT. However, in our study, Wexner constipation scores were not statistically correlated with the straining improvement. Thus, more clinical studies are needed to evaluate the influencing factors of WMT efficacy for FC-related therapeutic targets.

In our present study, diarrhea, abdominal pain, and bloating were the most common treatment-related adverse events observed during WMT treatment and the 24-week follow-up. These symptoms were mild and virtually self-limiting. Therefore, WMT has been generally considered a safe and well-tolerated treatment, as previously reported [9, 21, 42, 46, 51].

There are some limitations in the present study. First, this is a retrospective analysis of 63 patients at a single center without a control group for comparison. Ideally, a well-designed large randomized controlled trial would be better for evaluating the efficacy of a new treatment because of the advantages of randomization. However, patients with constipation are often receptive to seeking a range of available treatments, and thus it is difficult to guarantee that they will follow a doctor’s treatment restrictive regimens and obtain a full medication history. Second, colonic motility and anal manometry, which are helpful to categorize the type of constipation, were not performed in the present study. Therefore, we cannot compare the clinical efficacy of WMT among different constipation types and evaluate whether the motivation improves synchronously after WMT treatment. Third, in our study, we used patient-reported outcome measures [52], such as SCBMs per week, BSFS score, Wexner score, and constipation symptoms, to assess the treatment efficacy. Although patient-reported outcome measures have become increasingly popular in clinical practice and clinical trials, by the nature of being subjective, those outcome measures are prone to bias. Thus, measuring tools that conjunct with objective outcomes if available would be preferable. Fourth, microbial and metabolome analyses, which may clarify potential mechanisms of microbial remodeling ability of WMT on constipated patients for precise treatments, were not performed in the present study. Fifth, a placebo response cannot be ruled out in this retrospective study, and thus a clinical trial including a placebo control group is needed to determine whether the efficacy observed in the present study is due to an effect of WMT or a placebo response. Sixth, the change in diet is difficult to define and measure in a retrospective study, and thus its effect on the efficacy of WMT could not be assessed in the present study. Seventh, the intention-to-treat analysis was not performed in the present study, mainly because a considerable number (8 of 73) of patients were lost to follow-up. Thus, the efficacy of WMT in those lost patients remains unknown. A good cooperation between patients and doctors is very important to achieve a high follow-up rate with good clinical efficacy of WMT. Finally, the number (n = 63) of patients included in the present study was relatively small, which might not be sufficient to catch some efficacy or safety outcomes, such as “worsening of the symptoms” as observed in the present study. Therefore, future studies on the efficacy of WMT should take these limitations into consideration.

## Conclusions

WMT is efficacious in treating refractory FC-related therapeutic targets, especially straining, hard stools, and decreased stool frequency. The efficacy is increased with more than one course of WMT. However, a large-scale prospective study is required to further confirm the benefits of WMT for FC in terms of therapeutic targets.

## Data Availability

The datasets generated and analyzed during the current study are not publicly available due to none of the data types requiring uploading to a public repository but are available from the corresponding author on reasonable request.

## Abbreviations

FC: functional constipation
FMT: fecal microbiota transplantation
WMT: washed microbiota transplantation
SCBM: spontaneous complete bowel movements
BSFS: Bristol Stool Form Scale
TET: transendoscopic enteral tubing
IQR: interquartile ranges
OR: odds ratio
CI: confident interval
